# Promoting Sexual Health Knowledge through a Social Workers’ Mentorship Programme: A Study of Gay Young Adults

**DOI:** 10.3390/ijerph18115619

**Published:** 2021-05-25

**Authors:** Luis Miguel Dos Santos

**Affiliations:** Endicott College, Woosong University, Daejeon 34514, Korea; luismigueldossantos@yahoo.com; Tel.: +82-010-3066-7818

**Keywords:** gays, homosexuals, LGBT persons, men who have sex with other men, *Mentor Modelling Programme*, queers, sexual education, sexual minorities, Taiwan

## Abstract

Mentorship of counselling for men who have sex with other men and gay youths is understudied. The purpose of this study was to explore the effectiveness of how the *Mentor Modelling Programme* may increase the sexual health knowledge and practices of gay youths. As an expansion of a previous study with the application of the conceptual framework, this study mapped a sexual health promotion plan and the six-month-long *Mentor Modelling Programme* with the coordination of eight health and social care professionals and 40 gay youths. The researcher found positive and supportive feedback of how the *Mentor Modelling Programme* could increase sexual health knowledge, promote long-term relationships, and encourage referral of other vulnerable people. The results indicated two directions. The first solicited the perspectives of health and social care professionals and gay youths on how they would describe the relationship, application to, and experience of the *Mentor Modelling Programme* and second, assessed how this *Mentor Modelling Programme* influenced and changed the ideas and senses about counselling services and mentorship. This study reflected the current limited sexual promotion between traditional and inclusive sexual health materials. It further indicated the necessary concerns and areas of attention needed to upgrade the materials and host inclusive sexual health materials for both youths and adults in the communities.

## 1. Introduction

Effective sexual health education is a key element in any programme that aims to protect people from sexually transmitted diseases (STDs) [1], human immunodeficiency virus (HIV) [2], and acquired immunodeficiency syndrome (AIDS) [3], regardless of the gender, age, or sexual orientation of the target audience [4]. Homosexuality is not an illness, and nor is it a symptom of neurological or biological malfunction [5]. According to Blackwood, homosexuality is not a new concept in contemporary society. Same-sex relationship, behaviours, and activities were not uncommon in ancient human society. Homosexuality could be established by nature and nurture [6]. However, due to predominant social biases, many people still believe that homosexuality is ethically and morally wrong. Lesbian, gay, bisexual, and transgender (LGBT) youths are usually brought up in either heterosexual or single-parent families that are dominated by traditional anti-homosexual prejudices [7]. This is particularly true of East Asian families, communities, and societies [4].

Currently, most of the sexual health materials used in East Asian countries [8] tend to focus on heterosexual issues [9]. For example, most sexual health materials used in secondary schools are focused on the problems of underage pregnancy, STDs, HIV, and AIDS. Many East Asian parents, school leaders, and teachers do not encourage any sort of romantic relationships among secondary school pupils. Consequently, in many schools, sexual health education is largely limited to strictly theoretical and textbook-oriented learning [10]. However, although discouraged, teenagers cannot be entirely prevented from forming romantic relationships while at school. Indeed, due to the widespread use of social media applications by young people, this would be extremely difficult to achieve. Parents, school leaders, teachers, health promotors, public health specialists, doctors, nurses, counsellors, social workers, and policymakers, therefore, need to understand how to help these young people to protect their body and health [11]. However, it is important to emphasise that the predominant social and cultural biases in favour of heterosexual relationships mean that, even if there were a significant increase in the promotion of sexual health education, the materials produced would likely fail to serve the educational needs of sexual minorities young people [12].

Although health and social care professionals, social workers, counsellors, nursing professionals, and psychologists received appropriate training with patients from different backgrounds and issues, previous studies have expressed that many are not ready to work with the LGBT individuals [13], having limited practical and theoretical skills about issues in sexual health, sexual knowledge, and sexual promotion for these minorities [14,15,16]. Previous studies [17] advocated for upgrading awareness of these concerns in helping the LGBT communities by making an effort to seek out the backgrounds and needs of these individuals and groups [18]. One way to seek deeper information on LGBT individuals and groups, their concerns, confusions, problems, and issues is to train counsellors who went through a similar situation previously [19].

Another study [20] further indicated that Gay, Lesbian, Bisexual, Transgender, Intersex, and Queer (GLBTIQ) students’ national policy for counselling and protection plans is not being properly implemented. The results expressed that the government should establish effective plans, such as inclusive education, inclusive counselling, and supportive school environments with inclusive personnel, which could be useful to help GLBTIQ students during their teenage years. For example, counsellors with similar language ability, social-cultural background, previous histories, and skin colour may increase patients’ the sense of belonging and comfort. Therefore, beyond the current traditional ways of counselling skills and techniques, it is important to develop a new way of counselling practices in regard to the concerns of the LGBT communities [21].

The LGBT’s rights and movements have been exercised in the East Asian region, particularly in Taiwan, for more than three decades. One of the first advocators for LGBT’s right was managed by Mr. Chi Chia-Wei, the first activist for LGBT rights in Taiwan during the 1980s [22]. The conversation for LGBT rights and social equality was hosted in the legislative department in 1993. During this conversation, the organisations and non-profit groups for LGBT rights advocated that Taiwanese people, regardless of their gender, should enjoy equal rights, such as marriage and insurance. After rounds of conversations, discussions, and court cases, as a result, in 2017, the Taiwanese Constitutional Court advocated that rejecting two individuals of the same-sex or same-gender for the purpose of marriage is illegal based on the Judicial Yuan Interpretation No. 748 [23].

### 1.1. Purpose of the Study

Homophobia is one of the issues in the human communities which drive the negative attitudes toward the LGBT communities. As a result, many counselling services and family treatments do not have options for LGBT communities [6]. Therefore, a counselling scheme for the LGBT communities is essential. The purpose of this study is to examine the effectiveness of the relationship between health and social care professionals and gay youth, using the *Mentor Modelling Programme* [24]. Whereas heterosexual youths are provided with sexual health materials and counselling schemes that are specific to the education of their own sexual orientation, homosexual youths rarely receive appropriate sexual health information. Most of the current sexual health materials for young people tend to focus on the problem of early pregnancy [25], a focus from which homosexual youths do not benefit [26]. Instead of offering separate classes for homosexual-specific sex education (which may accentuate existing social bias), the researcher has developed a *Mentor Modelling Programme* (also known as the *Peer Modelling Programme*) that allows health and social care professionals to establish a relationship with young gay people in society. However, in order to better reflect the roles and positions of the participants and professionals in this study, the researcher changed the programme’s name to the *Mentor Modelling Programme.*

Although there has been increasing advocacy for the rights of individuals in sexual minorities in recent years, there remain many people in East Asian countries [8] who do not accept the legitimacy of homosexual relationships and behaviours [27]. Recently, in mid-2019, legislation was passed in Taiwan that allows same-sex marriage between homosexual couples [28]. However, although the government in Taiwan allows the registration of same-sex partners, many members of the public, including school leaders and public health professionals, do not understand how to handle the promotion of sexual health to members of the sexual minority communities [29]. In order to provide immediate help to sexual minority youths, the current *Mentor Modelling Programme* is intended for use by health and social care professionals. The study subsequently received feedback from health and social care professionals, who viewed the *Mentor Modelling Programme* positively [24].

The following research questions guided the research study:

1. From the perspectives of health and social care professionals and gay youths: how would they describe the relationships, application to, and experience of the *Mentor Modelling Programme*?
2. From the perspectives of health and social care professionals and gay youths: how would this *Mentor Modelling Programme* influence and change the ideas and senses about counselling services and mentorships after completion?

### 1.2. The Conceptual Framework for This Study

In 2020, a research study named “Promoting Safer Sexual Behaviours by Employing Social Cognitive Theory Among Gay University Students: A Pilot Study of a *Peer Modelling Programme*” [24] was published for the promotion of sexual minority sexual health issues. This study is an expansion of this research study. The previous study only employed social workers with concerns about sexual minority issues and sexual health promotions in Hong Kong. However, the current study invited additional health and social care professionals, including health specialists, nurses, and social workers, as the coordinators for participation.

### 1.3. Enhancement and Employment of the Previous Study and the Conceptual Framework

It is worth noting that this study is a further expansion of a previous study. First, as mentioned above, the previous study only invited social workers as the major coordinators for the pilot study, data collection, and examination for gay youths and the *Mentor Modelling Programme.* However, in order to increase the reliability and validity of this study, the current study further invited other health and social care professionals (i.e., health specialists, nurses, and social workers) who have the experience, interests, and focus on promoting sexual minority issues and sexual health problems in society. It is worth noting that these health and social care professionals have established knowledge and experience in sexual minority issues and sexual health promotion. Therefore, the employment of these professionals matches the nature of this study. They would also be the appropriate professionals to provide sexual health education and counselling for the youths of this study.

## 2. Materials and Methods

### 2.1. The Introduction of the Mentor Modelling Programme

The programme was undertaken in coordination with a group of health and social care professionals who work with sexual health promotion for sexual minority individuals and youths in Taiwan [27,29], where members of the public are influenced by traditional East Asian traditions and practices [8]. The *Mentor Modelling Programme* [24] is intended to provide mentor-level relationship(s), sharing, and mentor-level modelling partnership(s) to the target participants (i.e., gay youths). Due to the professionalism and registration problems, the researcher could only invite registered professionals in this study as professional mentors.

This study was a small-scale study for a potential larger-size counselling scheme for sexual minority individuals and potential minorities (e.g., single-parent, domestic violence victims, immigrants, children from orphanages, etc.). Before any larger-size schemes are established and contribute to the discussion, the researcher would like to test the performance and effectiveness of this *Mentor Modelling Programme* for a targeted group of minorities (i.e., gay youths) in society. In this case, gay youths were the samples. As a result, the researcher received positive opinions and feedback about the contributions and instructions of this *Mentor Modelling Programme* as an effective tool for solid and trustingful relationship(s) building and sharing from both health and social care professionals and participants (i.e., gay youths). The following part explains the progression and procedure.

First, the researcher introduced the *Mentor Modelling Programme* to eight health and social care professionals in Taiwan who work with sexual health issue(s) for the sexual minority communities. The health and social care professionals belong to three NGOs (Non-Government Organisations) that care about sexual health among sexual minorities and issues among youths in Taiwan. All received appropriate training in social work and psychological counselling at the university level with appropriate registration. Unlike the standardised and traditional sexual health materials with extensive materials for heterosexual behaviours and pregnancy, sexual health promotion for sexual minorities should not focus on the problems of pregnancy [25]. In other words, sexual health promotion materials should be tailor-made to meet the needs of this group of people.

Second, researcher [17] advocated that health and social care professionals, social workers, and counsellors should understand the patients’ backgrounds, lived stories, behaviours, and situations in order to provide the most appropriate treatments [13]. In order to meet these requirements, the health and social care professionals in this study must be gay men. Although Taiwan has recently allowed same-sex marriage, both health and social care professionals and youths received heterosexual-oriented sexual health knowledge from previous experience at the K-12 and university-level. Therefore, the researcher sought to use the sexual orientation (i.e., gay) as a solid relationship and connection engaging both health and social care professionals and youths in this mentor-based modelling programme.

Third, eight health and social care professionals agreed to join this study. Each health and social care professional was paired individually with five gay youths from 18–20 years old. Only the health and social care professionals had personal contacts and connections between the youths. The youths did not know each other. However, due to the limited population, the youths might know each other in general. The health and social care professionals took on the role of mentors and engaged in the youths’ daily lives and experiences. Both had close contacts with each other via social media chat (i.e., Line Social Media Chat) daily, and a bi-weekly face-to-face freestyle chat in a coffee shop, restaurant, or private space. Within the daily social media chat and bi-weekly face-to-face chat, the health and social care professionals always contributed to the homosexual-oriented sexual health promotion and material to the youths. During the six-month period, the health and social care professionals should meet at least ten times in a face-to-face chat with each youth. For the background of the health and social care professionals, please refer to Appendix A.

Fourth, the scope of this *Mentor Modelling Programme* plans to focus on personal relationship development, brotherhood, friendship, and peer-to-peer relationship instead of the traditional counsellor-to-patient relationship in the office environment. Therefore, the outcomes of this study tried to understand how these unique relationship(s) between each other may influence the sexual knowledge of our next generation(s).

In addition, the health and social care professionals created a portfolio for each youth based on their developments, progressions, behaviours, conversations, ideas, understanding of sexual intercourse, issues about STD and HIV testing, and further related topics [30]. Within the sharing and exchanging, the health and social care professionals always served as the mentors and friends (i.e., peers) of the youths, but not health and social care professionals. In fact, the relationship between mentors and friends (i.e., peer) always allowed both parties to exchange and share lived stories. Therefore, if the health and social care professionals take roles as health and social care professionals in this *Mentor Modelling Programme*, the youths may keep personal space and room in between them. The details of the procedures and steps were explained in the following section.

### 2.2. The Introduction of the Mentor Modelling Programme

#### 2.2.1. Pre-*Mentor Modelling Programme* Interview between the Health and Social Care Professionals and the Youths

A health and social care professional paired up with five different youths in a mentor modelling group individually. Both met in a private space where the youths shared and exchanged their sexual behaviours, sexual history, sexual preference, protections for sexual behaviours, knowledge about sexual health, and understanding of the sexually transmitted diseases (STDs), human immunodeficiency virus (HIV) [31], and acquired immunodeficiency syndrome (AIDS) as mentors and friends.

During the interview sessions, the health and social care professionals might conduct an audio recording of the conversation, complete a checklist for the elements, and create a portfolio. Before the end of the initial interview session, both must schedule the next interview or chat for the bi-weekly sharing and chat. However, due to the ad-hoc arrangement and unforeseen activities, the health and social care professionals should have room to re-arrange the procedure. However, the researcher always encouraged the health and social care professionals to follow the general guidelines.

#### 2.2.2. The Six-Month *Mentor Modelling Programme*

Qualitative researcher [32] indicated that interpersonal relationship(s) and trustfulness cannot be established within a short period. Particularly, personal experience, backgrounds, lived stories, sexual histories, sexual preference, protections for sexual behaviours, and knowledge about sexual health could not be changed overnight due to the social and cultural understanding [26]. Therefore, the six-month *Mentor Modelling Programme* would be appropriate as a long-term mentor-level relationship.

During the six-month procedure, the health and social care professionals might ask open-ended, semi-structured, and close-ended questions [33,34] based on the progress and development of the youths. Each youth has their own portfolio for their development. The health and social care professionals should indicate the progress of sexual behaviours, sexual intercourse, protections, and related information on the portfolio with dates.

Due to the uniqueness of the background, each health and social care professional should have their own counselling materials and tools (e.g., interview questions, portfolio, protocol, etc.). Although some researchers may argue that a uniform survey and materials should be employed in order to create uniform results, the open-endedness and freedom of design remained in this study. In fact, all personnel (i.e., professionals and youths) have unique and different perspectives, conceptions, and situations. Uniform materials were not appropriate due to the various elements and factors. Therefore, health and social care professionals had their own abilities to create and develop counselling materials for the youths. The researcher reserved that freedom and only provided the general direction and instruction to the health and social care professionals.

Some of the key terms for this *Mentor Modelling Programme* are the ideas of brotherhood, friends (i.e., peer), and common ground (i.e., gay individuals). Some may argue that traditional counselling theories and practices do not encourage the sharing of personal and sensitive information. However, one of the innovative procedures of this *Mentor Modelling Programme* is to establish a long-term relationship and trustfulness between each other. Therefore, based on the current *Mentor Modelling Programme*, both health and social care professionals and youths agreed on the arrangements and procedures of personal information, lived stories, and sensitive history sharing. Both expressed positive opinions and feedback for the improvement of sexual health, sexual promotion, and sexual knowledge.

#### 2.2.3. Post-*Mentor Modelling Programme* Interview with Each Youth

After the completion of the six-month *Mentor Modelling Programme*, health and social care professionals should arrange and polish the portfolio individually. The health and social care professionals arranged the Post-*Mentor Modelling Programme* interview with each youth individually in order to go through the overall performance, progression, and enhancement of sexual health knowledge. More importantly, the health and social care professionals asked the youth to complete a checklist/survey with appropriate sexual health information. The researcher and health and social care professionals analysed the increase of positive sexual health knowledge of these participants who completed this six-month programme.

Furthermore, the health and social care professionals should distribute the survey to each youth about their opinion and feedback of this *Mentor Modelling Programme,* the related progression, and the overall performance. The youth may return the survey to the health and social care professional immediately after completion or no more than one week after the interview via social media chat. It is worth noting that the youth should take a picture of the paper survey to send to the health and social care professional. Then, the health and social care professionals would collect and transfer to the researcher for data analysis.

#### 2.2.4. Post *Mentor Modelling Programme* Interviews and Focus Group Activity with the Researcher

The purpose of this study is to measure the effectiveness of this *Mentor Modelling Programme.* First, the researcher invited each health and social care professional for a rich, in-depth, and private individual interview of their feedback and opinion of this *Mentor Modelling Programme*. Each individual interview section [33,35] lasted from 121 to 148 min due to detailed sharing.

Exchanging and sharing the benefits, difficulties, understanding, and ideas about the *Mentor Modelling Programme* is also important between health and social care professionals as health and social care professionals are the users of this *Mentor Modelling Programme* in the future. Therefore, after the interview sections, the researcher arranged a focus group activity [34] with eight health and social care professionals together for sharing and expression. The focus group activity hosted 156 min.

#### 2.2.5. Member Checking Interview

In order to increase the validity of the study, the researcher further sent the analysed data information to each health and social care professional, respectively. All agreed with their data information. The member checking interview sections hosted between 56 and 77 min [32].

### 2.3. Data Analysis

Two groups of data information had been gathered: first, the sharing of each health and social care professionals and second, the survey from the youths. Firstly, the researcher listened to the recorded conversation and sharing (i.e., interview and focus group) from the health and social care professionals. After, the researcher transcribed the audio-recording into written transcripts for data analysis and reporting.

Secondly, the researcher also analysed the surveys from the gay youths in order to analyse the feedback from the participants. Therefore, the feedback from the two parties was greatly considered and understood.

The researcher analysed the health and social care professionals’ data information (i.e., interview and focus group) as a package for data analysis. To illustrate, the researcher analysed both interview sessions and focus group data information back-and-forth in order to categorise the meaningful themes and subthemes. On the other hand, the researcher also studied and analysed the youths’ survey responds for the reporting. After the researcher re-read and studied both data information (i.e., health and social care professionals, and youths) multiple times, the researcher could eventually categorise the meaningful themes and connections for reporting. As a result, the researcher reported the sharing and data information based on each individual. In order to indicate the findings, the researcher used the position and the data collection tool to indicate who shared, with labels such as “Health and social care professionals #1, Interview” and “Youth, #22.”

Based on the nature of qualitative research methodology, themes and subthemes were inductively categorised. The researcher employed the general inductive approach [36] to narrow down the large-size transcripts (345 pages) into first-level themes using the open-coding technique from the perspective of the grounded theory approach [37]. As a result, 18 themes and 29 subthemes have been merged. However, based on the recommendations from qualitative researchers, a qualitative research study should not have more than ten themes and ten subthemes. Therefore, the researcher continued to narrow down the first-level themes and subthemes.

**Figure 1:**
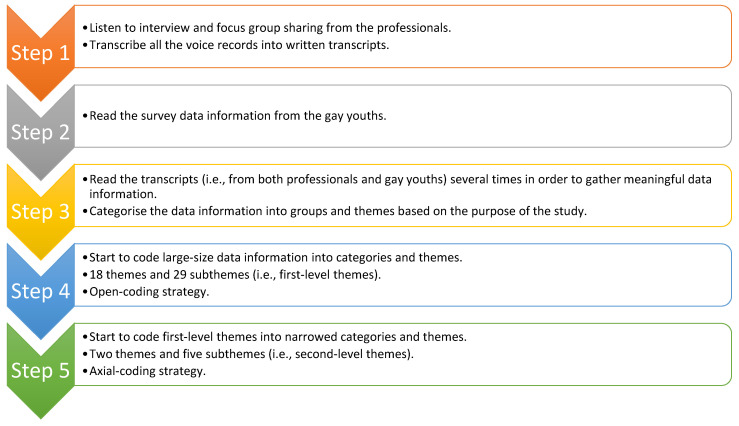
The data analysis procedure.

Qualitative researchers advocated that data information should be further narrowed down for meaningful themes and subthemes. Therefore, the researcher employed the axial-coding technique in order to categorise the second-level themes and subthemes. Based on the grounded theory approach, the researcher categorised the report with two themes and five subthemes. Figure 1 outlined the overall data analysis procedure.

### 2.4. Human Protection and Ethical Considerations

The protection of human subjects was the most important item for this study due to the nature of sexual health, sexual history, and other sensitive information sharing. The researcher made every effort to protect the participants’ (i.e., health and social care professionals and youths) identities. Therefore, in order to protect the identities and encourage all participants for sharing, the researcher assigned each participant by their role (e.g., Health and social care professionals #1; Youth #1). All subjects gave their informed consent for inclusion before they participated in the study.

The unsigned and signed consent forms and agreements, contact information, audio recording, transcripts, computer, and related materials were locked in a password-protected cabinet. Only the researcher has the rights to read the information. After the completion of the study, the related materials were destroyed and deleted due to the reasons for privacy.

All subjects gave their informed consent for inclusion before they participated in the study. The study was conducted in accordance with the Declaration of Helsinki, and the protocol was approved by the Ethics Committee of Social Welfare Team (2019/Summer-Fall/01). The study was supported by Woosong University Academic Research department.

## 3. Results

For both interview sessions and focus group activity, the health and social care professionals were asked the same questions about their experience of the study. Each was paired with the same number of participants with a similar sexual orientation, although the personal experiences and lived stories of participants were all different. Although Taiwan allowed same-sex marriage registration from mid-2019, this innovative change was not universally accepted [27,29]. It is important that NGOs and social caring organisations continue their work to overcome societal prejudices. The current six-month study, using the *Mentor Modelling Programme,* provided a long-term, solid, and effective tool that allows health and social care professionals to connect with vulnerable minorities [9].

During the data collection procedure, the health and social care professionals expressed the opinion that the *Mentor Modelling Programme* is a very effective tool and one that was previously unknown among practitioners in their field. Health and social care professionals, social workers, and counsellors of minorities’ sexual health had previously tended to employ passive counselling, an approach that meant that they were only able to provide help to a limited number of individuals. Nobody had imagined that the new model of gay-to-gay and mentor relationships could be a useful and effective method for the promotion of sexual health. Due to the tailor-made plans and procedures, none of the health and social care professionals expressed negative opinions about this *Mentor Modelling Programme.*

Based on the survey data information from the youths, none expressed negative opinions but positive and encouraging feedback and performances of this *Mentor Modelling Programme*. More importantly, a large number of surveys indicated that the youths would refer to other sexual minorities and vulnerable people to the services and centres for sexual health information and education. The following parts will outline the feedback and opinions from both health and social care professionals and youths. The findings were categorised into two themes and five subthemes. The details are listed in Table 1.

### 3.1. Social Status

Based on the written transcripts, the words useful, meaningful, and effective were found to have been used 188 times to describe the application of the Mentor Modelling Programme. Mentor modelling is one of the significant elements of the *Mentor Modelling Programme* [24]. The researcher required that participating health and social care professionals with a similar background always allowed the participants in their groups to share their lived stories with others that had a similar background. Many previous studies indicated that collecting in-depth and rich lived stories requires the building-up of long-term and solid relationships, as some shared experiences are sensitive, such as sexual histories and sexual background [4,16,24]. For example, heterosexual health and social care professionals may not understand why gay men desire to engage in particular activities. However, due to their similar background and previous experiences, gay health and social care professionals are better able to understand. Therefore, the participants did not need to explain *why* and *how* to the health and social care professionals. The health and social care professionals already knew the background. As one said,

*I knew why my patients wanted to go to the bar because I experienced that before…no need to say anything, I got you…we understand each other’s behaviour and thinking in the gay community…if you asked a female health worker to provide counselling to these patients, she would be really upset…*

(Health and social care professionals #2, Interview)

#### 3.1.1. Vulnerable Individuals: Gay Men with Mentor-Modelling Experience Sharing

When categorising the ideas about sexual health promotion and related materials, many participants indicated that the materials could not answer and meet the needs for sexual minorities. One health and social care professional, who used his lived experiences to teach his patients the importance of wearing condoms during sex, said,

*It is hard to convince men use condoms in the absence of any concerns about accidental pregnancies. Not using a condom is a mistake that is common to all men, regardless sexual orientation. But as a gay…worker or gay friend, I use my gay experience and sexual history to tell my patient why I use a condom every time when I have sex…we echoed each other as we are both gay…*

(Health and social care professionals #3, Focus Group)

Within the focus group session, other health and social care professionals immediately echoed such feelings about the efficacy of sharing lived experiences. Many said that they did not hesitate to share their lived stories as this was an effective way to build up relationships with their patients, provided that this sharing did not violate ethical guidelines. The six-month duration of the study provided the time needed to convince patients.

*It is hard to convince men…as many men have their own goals and purposes. But as a man, and as a gay man particularly, I knew what they were thinking…because I experienced that stage as well…changing from an open-relationship is hard for some people, but I knew how to do it…but if you asked a heterosexual man or woman for such conversations…at least I don’t listen to that heterosexual…not to mention my patients*

(Health and social care professionals #1, Interview)

Besides the health and social care professionals, almost all youths indicated that their peer and mentor (i.e., health and social care professionals) spent countless time and energy to transfer sexual health knowledge and ideas of personal development during the six-month duration, as one said,

*We are the abandoned groups of people in this community…no one really care about our life and existence. But my peer also encouraged me to start my own direction…I was lost…I don’t know how can I protect myself away from unsafe sexual activities…no one cared me at all…due to this Peer [Mentor] Modelling Programme …I know what to do now…*

(Youth, #22)

The above sharing indicated that the participants and other gay youths with a similar background face ignorance due to their sexual orientation in the community. All participants admitted that they also had met their long-term partners over the internet. Therefore, instead of asking their patients to stop using dating apps, they instead focused on teaching them ways of staying safe and protected. One said,

*I dated my boyfriend online. This is not uncommon as long as it is safe…I do not ask my patients not to use a cell phone…Instead, I used my experience as an example to my patients about how to engage in safer internet dating or further sexual behaviours…in this century, we cannot block them; we need to teach them with real-life or personal experience…*

(Health and social care professionals #5, Focus Group)

The lived experiences shared by the health and social care professionals were effective teaching methods, and both health and social care professionals and patients shared similar stories with each other. Although many parents discourage online dating, it is not possible to entirely prevent young people from forming relationships over the internet [38]. Therefore, the health and social care professionals focused on teaching young people methods of staying safe [8] (e.g., always using a condom).

#### 3.1.2. Vulnerable Individuals: Discrimination from Family, Friends, and Gay Communities

In cases where they do so, both health and social care professionals and patients said that they received many negative reactions. The current *Mentor Modelling Programme* was, therefore designed as a shelter-like mentor-level platform for the exchanging of knowledge and the sharing of experiences [24]. One health and social care professional, who claimed that this *Mentor Modelling Programme* allowed him to connect with gay patients who had hurt themselves due to social discrimination, said,

*A patient cut his arms and legs because his siblings and classmates laughed at his sexual orientation. As a gay man, I understood these feelings and I could share with him how to deal with the stress. I am sure heterosexual…workers cannot provide the same sort of effective counselling as they never experienced these discriminations…not only from society, but also from parents, siblings, and other family members.*

(Health and social care professionals #5, Focus Group)

Besides discrimination from family and friends, individuals within the gay community also discriminate against each other [39]. The *Mentor Modelling Programme,* on the other hand, proved itself to be an effective channel for the transfer of appropriate knowledge. One of the gay health and social care professional who employed it said,

*It’s not just gay people; everyone in East Asian communities compares their belongings with each other…many gay friends boasted about their sexual histories and the numbers of sexual partners that they had had…having multiple sexual partners should not be regarded as a positive thing …the Peer [Mentor] Modelling Programme always allowed me to use my experience as the tool to transfer appropriate knowledge to vulnerable youths*

(Health and social care professionals #2, interview)

Based on the above sharing, East Asian individuals tended to care about the viewing and opinions from other public members in the community [40,41,42]. Based on the sharing of their experiences, all health and social care professionals and patients were found to have experienced mockery and jokes about homosexuality in their classrooms and workplace [5]. One health and social care professional shared the experiences of his youth(s) after the successful establishment of the same-sex marriage law in mid-2019:

*All my patients told me that their teachers complained that same-sex marriage would destroy society…some patients were forced to disclose their sexual orientation in front of the classroom…some were asked to name negative points about homosexuality with their classmates. They received a lot of negative messages. The Peer [Mentor] Modelling Programme is an excellent and effective channel for the providing of appropriate knowledge and the correcting of the negative messaging that young people experience*

(Health and social care professionals #4, Focus Group)

Even though policymakers advocate for the rights of sexual minorities, many members of the public continue to discriminate against people based on their sexual orientation [4,5,43]. Gay youths are therefore left without any channels to express their concerns and feelings, since those who surround them in society consistently discourage homosexuality. Furthermore, many youths receive inappropriate knowledge from others. Undoubtedly, the coordination between the *Mentor Modelling Programme* [24] and the sharing of health and social care professionals’ lived stories, and personal experiences served as an effective channel for the transferring of positive sexual health knowledge and practices to gay youths.

#### 3.1.3. Referral of Other Gay Youths and Sexual Minorities

Referral is a common practice in the fields of health and social care as many individuals do not know how to seek help from health and social care professionals [44]. After several rounds of meeting and social media chats with the health and social care professionals, many youths recognised that upgraded sexual knowledge, sexual protection, and sexual promotion are important for their life. Therefore, they referred other youths with a similar background to the health and social care professionals. Before the *Mentor Modelling Programme* and sessions, these youths, however, did not believe in any sexual protections. It is important to note that the psychological beliefs and behaviours have been changed (i.e., for better).

Besides their current tested patients, all health and social care professionals were also helping a large group of sexual minorities. Some said that their tested patients consistently referred other gay individuals for questions, HIV tests, and counselling, and one said,

*Although I am currently working on 89 cases, I cannot reach all sexual minorities, particularly people without strong connections with other people…three patients referred my contact to some in-closet gay youths for HIV testing and condom use counselling…of course, I am glad…I can see the effectiveness and applications of this meaningful Peer [Mentor] Modelling Programme*

(Health and social care professionals #1, Interview)

Besides person-to-person referral, many health and social care professionals also referred some of the non-tested patients to other social caring organisations for further services. One said that many gay youths needed to have HIV tests, and that they could not provide these tests. Therefore, they could refer the patients to other NGOs and counsellors. One said,

*The HIV test is unique, and I cannot provide the test as I am not a medical professional…I can refer the patients to other NGOs. That is excellent as I can refer sexual minorities to others with this effective Peer [Mentor] Modelling Programme. Without this programme, these youths will be in danger…*

(Health and social care professionals #5, interview)

Four other health and social care professionals also said that they had to transfer some serious cases to the hospitals. For example, some youths used drugs and might be infected with HIV. Those referred patients were in-closet youths without connections with any health and social care professionals but who did know some of the tested patients. Therefore, the *Mentor Modelling Programme* served as one of the channels for referral.

*Some in-closet youth, not all, associated with drugs’ problems…I could see that the referral mechanism of the Peer [Mentor] Modelling Programme allowed me to reach additional vulnerable people in our community…If I can reach one more, I can save one more life…thank you for this Peer [Mentor] Modelling Programme*

(Health and social care professionals #1, Focus Group)

In short, all the health and social care professionals gave positive feedback on the *Mentor Modelling Programme*. They said that the programme allowed them to use their personal experiences and lived stories as the tools to transfer appropriate knowledge to other vulnerable sexual minorities. Health and social care professionals could create additional tailor-made portfolio and counselling works beyond this programme for the potential larger-size application in the future.

### 3.2. Long-Term Relationships

#### 3.2.1. Trustfulness

The following two subthemes outline how the *Mentor Modelling Programme* allowed them to build up the mutual trust and respect that was required for the programme to work. All the health and social care professionals involved expressed satisfaction in the effectiveness and results of the *Mentor Modelling Programme,* saying that it allowed them to build up life-long relationships with tested patients and made it easier for them to reach referred patients. This was something that they had never experienced while using traditional counselling services. In particular, this was the first time that they had been able to build up connections and networks within the gay community. One said,

*Young people will not have the same interests as myself, a middle-aged gay man…so if I entered the gay youth’s community, people would believe that I was looking for a one-night-stand…but with this effective and meaningful Peer [Mentor] Modelling Programme, I can use my middle-age knowledge and role to reach the inner world of gay young people…if we cannot understand the behaviours of the patients, we cannot provide effective counselling to the patients…but as my patients continued to share stories to me even after the completion of the programme, I can see that the trustfulness is there…*

(Health and social care professionals #1, Focus Group)

The ideas of the long-term relationship were not shared by the health and social care professionals. Some youths also indicated that the long-term relationship was meaningful and effective to their sexual health and personal enhancement, as one youth said,

*I enjoy the friendship with my peer…he is my mentor, my big brother, my friend, and a part of my family I believe…we met each other every month and every day* via *social media chat…I shared my life to him and he also came to my workplace for visiting…it is not only a social welfare programme…but really a friendship and relationship…*

(Youth #5)

Some believed that the sharing of real information was the key element in this programme. Many said that during the first two months, some youths might not share accurate and real information as the health and social care professionals were strangers. However, all claimed that the youths could feel the efforts and peer or mentor-exchanging ideas from the health and social care professionals via the *Mentor Modelling Programme.* Trustfulness was eventually established. One health and social care professional said,

*Some youths might share fake information in the first meeting or during the first several months…but we really shared real sexual health knowledge and even personal histories with them as the materials…they can feel that I or we (i.e., health and social care professionals) are here with them and helping them…a human being is not a machine, they eventually understood…*

(Health and social care professionals #2, Focus Group)

In short, all believed that the relationship between the *Mentor Modelling Programme* and the establishment of trustfulness was obvious and effective. Particularly, the duration of the *Programme* allowed both parties to establish trustfulness through continual sharing and exchanging of sexual health knowledge [45,46]. The friendships and mentorships that were established during the programme were life-long and solid, and this helped patients to continue their healthy lifestyles and encouraged them to refer others [47].

#### 3.2.2. Respectfulness, Brotherhood, and Life-Long Changing

Besides the establishment of trustfulness, both health and social care professionals and patients expressed different levels of respectfulness for each other. For example, the health and social care professionals could feel the respectfulness from their patients due to their long-term efforts, their sharing of sensitive information, and their provision of 24/7 support [45]. One of the successful outcomes for this *Mentor Modelling Programme* was the relationship and life-long changing. Many patients indicated that the health and social care professionals are their “brother” or “peer” instead of health and social care professionals without a solid relationship. In other words, the patients recognised the health and social care professional(s) as one of the important personnel in their life. Such relationships, in fact, are hard to establish among vulnerable personnel and sexual minorities in the East Asian region. Eight health and social care professionals, who shared in the focus group that they gained many younger brothers as a result of this *Mentor Modelling Programme,* said,

*East Asian people like to build up brotherhoods with close friends and people who really know each other. Due to this meaningful Peer [Mentor] Modelling Programme and its six-month long duration, I really gained a lot of young brothers in my life, which is very rewarding*

(Health and social care professionals #4, Focus Group)

In short, the outcomes and effectiveness of this *Mentor Modelling Programme* always continued after the end of the programme. The results and findings indicated that due to the friendship and brotherhood between the health and social care professionals and patients, many had established the trustfulness and the channels to exchange appropriate sexual health knowledge and materials. All health and social care professionals recommended that this *Mentor Modelling Programme* should be contributed to other channels and groups for professional counselling services [9,30,43].

## 4. Discussions

### 4.1. The Social Status, Relationship, and Experiences

During the data collection sessions, as reflected by a previous study [48], all insisted that more than half of current sexual health promotion materials do not meet the needs of gay men (e.g., the amount of attention devoted to underage pregnancy). In order to fill the gap in the coverage of essential sexual health topics, the health and social care professionals used their own experiences and lived stories as the materials for educating their patients [49]. In line with McCarty-Caplan [30] on the topic of how to promote safer sexual behaviours, many expressed the opinion that having a single sexual partner could increase safety, and health and social care professionals shared this advice with their patients [33]. A previous study [34] indicated that the relationship(s) could not be established within a short period. Therefore, under the current East Asian social and cultural background [47], the health and social care professionals should spend time for each in order to establish a closed relationship.

A previous study [7] indicated that social media and cell phone applications are some of the most common channels used by gay men for dating. Both homosexual and heterosexual individuals arrange one-night stands over the internet. Instead of discouraging or preventing gay men from using these dating apps, health and social care professionals provided counselling about safer sex [44]. Mentor modelling and mentor-level sharing and exchanging played important roles in this study [30]. Many health and social care professionals claimed that discrimination is a serious problem in both the heterosexual and homosexual communities in Taiwan [27,28,29,50]. Some claimed that gay individuals like to compare and boast about their property, sexual partners, unsafe sexual practices, and even the use of drugs. This can lead to some young people being influenced by the negative behaviours of other gay individuals. Although the previous studies indicated that counselling services could increase the sense of belonging of individuals in the community, such senses cannot change how people judge each other [4,5,9]. Moreover, discrimination comes not only from family members, friends, and members of the gay community. Teachers at schools always make fun of homosexual and gay topics in their classrooms. Although same-sex marriage and related rights are protected by law, it does not mean that social taboos and bias are eliminated [23].

The referral is a common practice in the fields of health and social care as many individuals do not know how to seek help from health and social care professionals [4]. The *Mentor Modelling Programme* encouraged their patients to refer other gay individuals with a similar background to join and consult for counselling and HIV testing services. Gay youths tended to listen to other people with a similar background, in this case, homosexuality. Therefore, the mentor-level platform provided effective channels for transferring and exchanging knowledge [10]. More importantly, the effectiveness of the programme led to additional referrals to other sexual minorities who also needed help from health and social care professionals [20].

### 4.2. Change the Ideas and Senses about Counselling Services and Mentorship

Duration is a significant element of this study. In the field of health promotion and counselling, established and solid relationships and friendships need time to properly develop [17,30,51,52]. This study focused on imparting sexual health knowledge employing sharing sexual histories and preferences. Still, without solid relationships, individuals would have been far less likely to share such sensitive information with strangers. Therefore, the six-month duration of the *Mentor Modelling Programme* allowed both health and social care professionals and patients to establish long-term relationships that might even last for life.

In the fields of health and social care, trustfulness is essential [31,53]. Patients share a lot of sensitive details with their professionals, and these details are particularly sensitive in the case of sexual minorities living in a biased society. All the health and social care professionals involved expressed satisfaction in the effectiveness and results of the *Mentor Modelling Programme,* saying that it allowed them to build up life-long relationships with tested patients and made it easier for them to reach referred patients. It is worth noting that the collectivism [41,42] and the East Asian perspective about trustfulness highly influenced the effectiveness and the relationship(s) between the two parties. With the reflection of the *Mentor Modelling Programmes* and the previous study [45], it is worth noting that the peer-to-peer or mentor-to-peer relationship(s) worked for the current groups of participants (i.e., gay youths).

The transferring of appropriate and positive information about sexual health to sexual minorities is the key aim of this study. Besides the establishment of trustfulness [47], both health and social care professionals and patients expressed different levels of respectfulness for each other [54,55]. This was largely because of the friendship and brotherhood between the health and social care professionals and patients that had been established during the programme’s six-month duration [56]. More importantly, this research study and the *Mentor Modelling Programme* echoed the ideas of East Asian perspectives [57] and the traditional practices of collectivism for East Asian patients. Many believed that, unlike standardised and traditional treatments in which many health and social care professionals only view their patients as customers, the unique relationships that arose from the *Mentor Modelling Programme* allowed both patients and health and social care professionals to gain a thorough understanding of each other [20].

## 5. Limitation and Future Research Direction

Every study has limitations. There are six limitations to this study. First, the study could only collect and invite data information from gay youths. Due to the nature of a small-size pilot study, this study tended to take gay youths as the major participants. However, other sexual minorities, such as lesbians, bisexuals, and transgender individuals’ feedback and opinions were missing. Therefore, future research studies may expand the concerns for these individuals.

Second, the current study captured the data information and application from gay youths in Taiwan. However, gay youths in other regions and countries, such as Japan, South Korea, China, with a similar background and cultural perspective, may share the benefits of this *Mentor Modelling Programme.* Therefore, future research studies should employ the *Mentor Modelling Programme* and capture the information from regions and countries with a similar background for a comparative study.

Third, as the *Mentor Modelling Programme* is established as a guideline for the professionals and youths to follow, many of the counselling materials and tools were designed and managed by the professionals. Therefore, some may argue the overall performances and results of the *Mentor Modelling Programme* are limited. After the completion of this study, the policymakers, organisational leaders, researchers, and government leaders should take this study as an opportunity and blueprint to reform and polish the current sexual health promotions and materials for sexual minorities in the society.

Fourth, the researcher should introduce a Knowledge, Attitudes, Beliefs, and Behaviours (KABB) survey to understand the overall developments and improvements of the youths (i.e., about the sexual knowledge and improvements). However, as this study focused on the satisfaction and overall arrangement of the *Mentor Modelling Programme*, a different scope was the focus. Therefore, for future research and further studies, researchers should employ the KABB survey, which may allow the health and social care professionals to have a better understanding of how to improve and develop their practices.

Fifth, some researchers may argue that the study may be conducted as a controlled group arrangement. However, as this was a small-scale pilot study without additional resources, staff, and individuals, the researcher sought to understand the overall performance of this *Mentor Modelling Programme* before additional development. More importantly, as homosexuality is still a taboo in the East Asian region, not all homosexual individuals are willing to join this study. Therefore, the current arrangement has been the greatest capacity based on the current social situation. However, in the future, if additional resources are allocated, large-size tests and evaluations can be exercised.

Sixth, as the current study focused on the experiences and the feedback of the *Mentor Modelling Programme*, the concerns from the general public members are missing. Therefore, future research may expand the understanding and directions to the homophobia in the community.

## 6. Conclusions

First, this study contributes to the understanding of how the *Mentor Modelling Programme* could influence the sexual health and attention of sexual promotion plans for sexual minorities, particularly gay youths in the East Asian context and environment [8]. Although Taiwan is the first place for sexual minorities to establish the relationship, it is not hard to believe that discriminations and social biases existed in different parts of society and communities [27,28,29,50]. The research study can also further reflect the concerns to other countries and regions in the East Asian environment.

Second, the shared information from this study reflected the current limited sexual promotion between traditional and inclusive sexual health materials. The study indicated that concern for and attention to upgrading the materials and hosting inclusive sexual health materials for both youths and adults in the communities is necessary.

Third, as the results of this current study were positive, the researcher will coordinate with other NGOs and appropriate leaders to promote the effectiveness and outcomes to some policymakers and departments for research funding and application. More importantly, the procedures and applications of this *Mentor Modelling Programme* may be further contributing to other minorities with exclusive life experiences.

Last but not least, the Department of Health, health and social care experts, researchers, educators, school administrators, policymakers, and health specialists should take this study as the opportunity to reform and polish the current sexual health materials and establish new policies to help people with special and non-traditional needs in our society.

## Figures and Tables

**Table 1:** Themes and subthemes.

Themes and Subthemes		
3.1.	The Social Status	
	3.1.1.	Vulnerable Individuals: Gay Men with Mentor-Modelling Experience Sharing
	3.1.2.	Vulnerable Individuals: Discrimination from Family, Friends, and Gay Communities
	3.1.3.	Referral of Other Gay Youths and Sexual Minorities
3.2.	Long-Term Relationships	
	3.2.1.	Trustfulness
	3.2.2.	Respectfulness, Brotherhood, and Life-Long Changing

## Data Availability

Data available on request due to restrictions of human subject protection.

## Appendix A

The following information is the background of the health and social care professionals. All of these health and social care professionals are registered professionals who can host counseling sessions to the participants (i.e., gay youths) in this study. All are holding a valid registration in Taiwan.

**Table A1:** Background of the health and social care professionals.

Name	Academic Programme	Year of Experience
Health and social care professionals #1	Social Work	10+
Health and social care professionals #2	Social Work	10+
Health and social care professionals #3	Social Work	10+
Health and social care professionals #4	Social Work	15+
Health and social care professionals #5	Social Work	15+
Health and social care professionals #6	Social Work	15+
Health and social care professionals #7	Nursing and Social Work	15+
Health and social care professionals #8	Social Work and Counselling	15+
